# Selections and Abstracts

**Published:** 1900-09

**Authors:** 


### *SELECTIONS AND ABSTRACTS.*

### The Treatment of the Vomiting of Pregnancy.

The so-called pernicious vomiting of pregnancy is a condition which, like postpartum hemorrhage, is fortunately not very commonly met with, and yet, like the hemorrhage that we have just named, when we do meet with it, it is so grave and harassing a condition that it forces itself upon the attention of the physician in such a way as to leave memories of it for many days to come. Our attention has recently been called to this important subject not only by personal experience, but also because of two papers which have appeared in a recent issue of the *Boston Medical and Surgical Journal.*

It is rather important in beginning to consider this subject that a clear distinction be made between the pernicious or persistent type of vomiting which we are now discussing and the ordinary nausea or morning vomiting of pregnancy which so many women experience. The latter condition is, of course, disagreeable and annoying, but usually is not sufficiently persistent to distinctly impair the woman's strength and vitality. In the graver form, on the other hand, there is a constant tendency from beginning to end to sap vitality and to produce a condition of collapse and shock. There can be no doubt that it is the duty of the physician in a great majority of cases of this character to temporize for at least the first few days, or until such time as it becomes evident that the woman is being distinctly injured by the persistency of the vomiting. He should temporize, first, because ordinary medical measures may quiet reflex irritability, because the vomiting may not be due in reality to the pregnancy, and finally, because it is desirable to bring the woman to full term without in any way interfering with the physiological process which we call "pregnancy," and destroying the life of the child yet unborn. Curiously enough, there is even at the present time distinct difference of opinion between eminent authorities as to the duty of a physician in the presence of vomiting of the pernicious type. In one of the articles in the *Boston Medical and Surgical Journal* that we have quoted, written by Dr. Twombly, he quotes the following authorities as being for and against radical interference in severe hyperemesis:

"Klein says: 'In hyperemesis of the third degree the artificial induction of labor is occasionally required.' Bacon says: 'Induction of abortion is never indicated.' Leclerc states: 'Good results . . . by simple cauterization of cervix; this measure is far superior to artificial abortion.' Lusk says that uncontrollable vomiting is a rare event, but when it occurs 'there remains as an ultimate resource the artificial induction of abortion.' A. F. Currier, New York, says in review: 'Finally there remains the emptying of the uterus as a last resort. It should only be done after careful deliberation and with the approval of skilled counsel.' Edward Reynolds justly remarks that the solution of the question depends in 'a certain degree upon the religious beliefs of the individual family, and upon their estimate of the relative value of maternal and fetal life. Among Protestant physicians in Protestant families it is generally considered the best practice to advocate abortion when all other treatment has failed, but patients are constantly lost by over-conservatism in the most skilled and experienced hands.' Gardner says: 'As regards the ultimate procedure of emptying the uterus, the general tendency is to delay too long the operation, one which in itself is not without danger.' Jewett states: 'Evacuation of the uterus is often too long delayed.'"

Delay and its dangers are emphasized by these last three men. Delay and its dangers are what Dr. Twombly emphasizes to us, and he enters his plea for earlier interference, before the patient's courage is gone, her vitality exhausted, and her system in the worst condition to withstand the shock.

The indications for emptying the uterus are: (1) Inability of retaining any food taken by the mouth; (2) intolerance of rectal enemata; (3) more or less albuminuria; (4) progressive emaciation; (5) headache constant; (6) frequent and feeble pulse; (7) a certain apathy of the patient.

It is important to remember in dealing with this class of cases that the vomiting may arise from some displacement of the uterus, which when corrected will cause immediate relief to the symptoms; that it may be due to uremic poisoning complicating the pregnancy; and again, that it may be due solely to reflex irritation of the vomiting center, and the most careful examination of the pelvic contents will fail to reveal any abnormality in position. It is in this latter class of cases that the physician should be most cautious in trying all legitimate medicinal measures before going on to any more radical interference. If, however, the administration of remedies which tend to quiet such excitement of reflex action fail, and it is evident that the patient is rapidly losing strength, and even that her life is in danger if the vomiting persists, then it is surely the duty of the medical man in attendance to empty the uterus under strict aseptic precautions. Under these circumstances, in the vast majority of cases if the vomiting is really due to the pregnancy, relief will at once follow. We believe that the decision to perform this operation is an exceedingly important one, first, because it interferes with pregnancy and with the life of the unborn child, and secondly, because in certain cases it is undoubtedly the only thing which will preserve the mother's life.

It is certainly a mistake to permit the vomiting of pregnancy to go on for so long a time that what may be called "the vomiting habit" is established, and the patient's strength is so exhausted that she is unable to recover even if the uterus is emptied, or is gravely shocked by the administration of the necessary anesthetics and the emptying of the womb. Even in cases which seem at death's door from the causes which we are discussing, we believe that the operation should be performed, since, in some cases at least, if they are as near death as we have described, recovery will occur. Such patients should be supported during the administration of the anesthetic and afterward by the use of normal salt solution given by hypodermoclysis; should have heat applied externally, and should be treated in every way as would be treated an ordinary patient suffering from surgical shock. By this means a crisis can often be passed over, and in a few hours after the uterus is emptied small quantities of concentrated and easily digested food may be given and retained.

Very recently we have found in a number of cases of persistent vomiting, and in one very noteworthy case of pernicious vomiting of pregnancy, that the patients did better upon starchy foods than they do upon meat broths and milk. Ordinary barley, rice, or oatmeal gruels were prepared, and to them was added a little liquid taka-diastase. Sometimes only as much as one or two teaspoonfuls of the gruel with a fraction of a grain of taka-diastase was given every hour or two until the stomach was able to retain more. In other instances where the patient was able to take a somewhat larger dose of the gruel, the taka-diastase was either given in liquid form or in capsules immediately after the gruel had been swallowed. The advantage of this treatment exists in the fact that the taka-diastase very rapidly converts the starch into such a form that it can be readily assimilated and utilized, and it does not lie in the stomach long enough to undergo any deleterious change. We do not believe that it is possible to give milk and animal broths without producing in a certain number of cases gastric indigestion and a foul tongue. On the other hand, we are well aware of the fact that what succeeds in one patient will not succeed in another, and that there are doubtless cases in which the various gruels that we have named would not do as well as peptonized milk or beef tea. It is our belief, however, that the profession usually employ milk and beef tea to the exclusion of gruels in the treatment of obstinate vomiting, and we are desirous of calling attention to these starchy foods under such circumstances, provided that their digestion and absorption are practically assured by the administration of the taka-diastase that we have named. Twenty or thirty minims of taka-diastase may be given with each small quantity of gruel, and if large quantities are given, at least two drachms of taka-diastase should be used. If the dry taka-diastase is employed, from one to three grains may be given. It is perfectly true that smaller quantities than those named digest larger quantities of starch, but on the other hand it must be remembered that our whole object is to get rapid digestion and absorption before the stomach ejects its contents, and therefore it is best to give an excess of a harmless substance like taka-diastase, rather than too little.—*Therapeutic Gazette.*

### THE LEGAL PREVENTION OF TUBERCULOSIS.*

Ex-Judge Abram H. Dailey, a former president of the Medico-Legal Society, and a diligent student of the science of medical jurisprudence, when asked to send his views in reply to the following question: "What has been, in your opinion, the most notable advance in that branch of forensic medicine, with which you are familiar, during the nineteenth century?" replied as follows:

My answer is, that, in my opinion, that which has prevented the inception and spread of disease, through sanitary laws, based upon a knowledge of the causes of disease and the removal of the same, is the most notable.

The whole civilized world is interested in that subject. Medical and legal minds have everywhere been directed to it, and through their efforts legislative action has been taken, and thereby all civilized people enjoy a sense of security against the ravages of contagious diseases that they have never before experienced. The future is sure to increase this safeguard.

Dr. Henry B. Baker, secretary and general executive manager of the Michigan State board of health, of Lansing, Mich., in response to the same question, said:

The most notable advance in forensic medicine, in the nineteenth century, is the aid which the law gives to the restriction of that disease which causes most deaths.

One reason why this is true is because that disease which causes most deaths is now known to be a preventable disease. Another reason is that it destroys our people at the ages when, except for this disease, most of them are in the prime of life; most of them are at an age when the cost for their rearing and education is at its maximum; at least half of those who die of this disease are between the ages of twenty and forty-five years, when their value as wealth producers is greater than at any other period of age. This one preventable disease causes the deaths of more than one hundred thousand persons in the United States, in every year. Considering the fifty thousand between the ages of twenty and forty years, if we estimate their value as wealth producers as equal to that of an average slave before the war ($1,000), and considering the losses incident to the premature deaths of another fifty thousand persons, and incident to the long-continued sickness from this disease, of the many thousands who finally recover enough to die of other diseases, it is a low estimate that this country loses thereby one hundred million dollars per year.

Without the aid of the law, the restriction of this most important dis-

*Read by Clark Bell, Esq., LL.D., of New York, secretary and treasurer of the American Congress of Tuberculosis, before the Medico-Legal Society at its June session, 1900.

ease cannot be systematically and thoroughly entered upon; for this work, *notification of cases* must be required to be given to local health officials.

In 1893 the Michigan State board of health passed a resolution, "That hereafter, consumption (and other diseases due to the bacillus tuberculosis) shall be included in the official list of 'diseases dangerous to public health' referred to in the law requiring notice by householders and physicians to the local health officer as soon as such a disease is recognized." The requirement has not been fully complied with throughout the entire State, but there is reason to believe that partly in consequence of this action the death-rate from that disease in Michigan has been reduced by rather more than one-tenth, and that the sickness from that disease has been decreased by a larger proportion. The success which has followed the imperfect action in Michigan, and in other states and countries, is good ground for a continuance in the belief which prompted the action—the belief that tuberculosis is a disease which can be restricted as soon as beneficent laws for this purpose shall be fully operative.

The medical profession has in the past, with that great conservatism, which seems to have been the inspiring thought of the medical mind of the nineteenth century, doubtless due to its methods of medical education in the American States, confined itself to the treatment of consumption.

It has been only very recently that a very few of the more courageous among medical men have declared publicly that consumption could be cured.

They have done this at the greatest risk, and as a rule have been denounced by their brethren as quacks and impostors.

Still, among the more advanced thinkers among medical men, the impression gains ground that it is a curable disease, while a majority of medical men to-day doubtless regard it as certainly in the class of diseases where preventive means are justifiable.

Advanced thinkers among medical men unite with great unanimity in declaring that it is infectious, and that the bacillus of tuberculosis is well determined and certainly ascertained and known, but this belief is believed to be far from universal, even among medical men.

That it is a proper subject for legislation, for the use of all proper means to prevent or arrest its spread or increase, is now very generally conceded, and the Michigan legislation, and the statistics from that State show that similar legislation in other States, and still more radical measures, might be of great public good in arresting the alarming spread of "this scourge of the human race."

This brings the question directly into the domain of medical jurisprudence.

How far can legislation be of service in affecting or securing beneficent results to mankind, in diminishing the volume of the disease, or in arresting its terrible advances?

The question has become one of profound and intense interest. It is the uppermost question of the hour. The recent Congress of Tuberculosis in Italy, the great interest in London and the American States made it advisable, in the opinion of the officers of the Medico-Legal Society, to bring it into prominence, and the organization of the American Congress of Tuberculosis was formed, as the results of its opening sessions on this theme. Its work is regarded simply as an introduction to the scientific world of the discussion of the subjects introduced.

An organization was perfected, and the collaboration of all who take an interest in the movement was solicited.

---

### FIREMAN'S CRAMP.*

The subject of "Fireman's Cramp" is one rarely heard of in medical journals, and but lightly touched upon in text-books. When we consider the conditions surrounding a fireman's or coal-passer's work, we must think that anything that may serve to mitigate his condiion will surely be appreciated.

As surgeon on an ocean steamer, my duties brought me in close contact with these laborers of the ship. Interesting psychologically at all times, they were more so when disabled by accident or disease. My attention was strongly drawn, during my earlier trips, to the condition known as "colic," or "cramp." The acute pain, the extreme prostration, the blood-shot eye, pressing thirst, labored breathing, irregular or weak heart action, and agonized expression

---

* Prize paper in literary contest of Merck's Archives, by Willis Cummings, M.D., Brooklyn, N. Y.

all denote a startling condition. My experience has been gained solely on steamers running to the tropics, the time of a round voyage being from three to four weeks.

The men in question, with but rare exceptions, are drinkers, and come aboard to begin a trip showing to a greater or less degree the effects of the misuse of alcohol. They immediately put themselves in a position to feel the worst effects of their imprudence by assuming their duties in the fire-room, where, in winter, they are fanned by the icy air coming through the ventilators, and in summer, especially in calms or following winds, they are in the vestibule of Satan's domain. Only one who has stood before the furnaces of a large sea-going steamer can realize the fatiguing situation of these men, who, during a period of four hours, shovel in tons of coal and remove and cool piles of ashes. They, perhaps, have been on "short rations" for a day or two previous to sailing, substituting for cheap food cheaper whisky. For several days after coming aboard their stomachs revolt at food, and only by constant urging on the part of the doctor, if there be one on board, will they perhaps take a bowl of hot soup or other nourishing aliment. There apparently is a studied indifference or actual ignorance on the part of the engineers in the matter, as they fail to see that what the men want is proper food rather than alcohol. They are not allowed to stop work until they drop, and the justice of this is that they have no business to jeopardize the interests of their employers by anything that will render them unable to perform their duties. Human nature is the same in the forecastle as in the officers' quarters. Personal appetites dominate impersonal interests at times in both places.

Finding the stock remedy to be "fireman's cramp mixture," a concoction resembling "Sun cholera mixture," inadequate to allay the condition, and leading, if used freely, to gastric disturbances and constipation, I sought for a substitute, and the one here described is the one decided upon as being the best. Under this method I found no case of colic to occur even in the hottest weather of the Gulf of Mexico, and it *is* hot there often. The men, without exception, did better work, and were less exhausted when coming off watch; their appetites were good, their spirits were cheerful, *and there was less desire for alcoholic stimulants* than after the anodyne methods.

My starting proposition was, that on account of the primary debility induced, directly or indirectly, by excesses, aided by the loss of fluids and solids in the profuse sweating brought about by heat and muscular action, there was a lowering of the vital force which, acting on nerve centers, produced a temporary or reflex paralysis of part of the abdominal contents, and also a spasmodic condition of the muscles, which, extending to the limbs, produced the characteristic contractions and painful seizures. Acting on this theory, I set about estimating the amount of moisture eliminated, and so far as possible, in a crude way, the amount of solids thrown off by skin and kidneys. Taking the whole number of hours per day, per man on watch, as twelve, it was estimated that they drank a certain quantity of water during the week's work. Estimating the loss of water and solids during the same time, the two were balanced and the following formula and conclusions were arrived at:

To one gallon of water were added

| | |
|---|---|
| Calcium Phosphate | 256 grn. |
| Sodium Phosphate | 256 grn. |
| Potassium Phosphate | 256 grn. |

with an excess of ortho-phosphoric acid.

For the use of nine men in four hours four ounces of this solution were put into nine gallons of water, containing oatmeal and ice if available, each man drinking approximately a gallon of water during the watch. The quantity of the phosphate solution is about three and one-half drams taken while on duty. The effect was immediate, and "phosphose," as they named it, was in demand daily. After some months I gave up the experiment, as it was conducted at my personal expense. I have used it occasionally since in conditions approaching "colic" with excellent results.

It was observed during the period of its continuous use that at times the men would not use "phosphose" for several watches. When asked why, they said they did not feel the need of so much of it, as the previous quantity had seemed to brace them up permanently. While the results were all desirable and satisfactory, they remain valueless unless applied to the amelioration of the condition of these "toilers in hell," as they have been aptly called by one who has worked among them.

It is a lamentable fact to the traveling public, as well as the hundreds of officers, engineers, sailors, fireman, stewards, and waiters, that but two American steamship lines out of New York carry regularly commissioned medical officers. No one who has sailed to tropical seas on a doctorless ship will fail to say that they have seen the necessity for medical attendance when no efficient help was near. While the law protects the steamship owners, their crews sometimes undergo great hardships in consequence of inadequate attention when sick or disabled. It has occurred to me that if the formula I have devised could be put into convenient shape, and its merits presented in a suitable way, they might adopt it. I am sure that the men who need it would take to the preparation kindly if once they could get started in its use. There are thousands of fireman and coal-passers sailing to every sea, and while many are not affected with "colic," many more are, and, like sunstroke, it sometimes attacks those who least expect it.

The running time of a ship depends very largely on the capability and endurance of the firemen. If the possibility of collapse is eliminated wholly or in part by a simple preparation, used as a pleasant beverage, the men themselves, their employers, and the business public will receive direct benefit.

In conclusion, I may say that by the use of this mixture I have relieved present conditions, avoided possible troubles, dispensed with "colic" and "cramp" mixtures and alcohol, and have stimulated a natural desire for food, and in consequence got more and better work from a class of men whose labors and pains are but little understood by those who know only the saloon deck life on the sea.—MERCK'S ARCHIVES.

---

### PRURITUS ANI, WITH ESPECIAL REFERENCE TO ITS LOCAL TREATMENT.

Adler writes in the *Philadelphia Medical Journal* of May 12, 1900, on this subject. He states it is important to see that the patient has a daily evacuation of the bowels, and, if necessary, medicines are to be used for this purpose.

In all cases more or less varicosity of the hemorrhoidal vessels exists; at all events, he is in the habit of seeing the patient daily for a time, and he employs an injection into the cavity of the rectum of 1 to 2 or 2½ drachms of the following prescription:

Fluid extract of hamamelis.......................1 fluid ℥;
Fluid extract of ergot, ........................2 fluid ʒ;
Fluid extract of hydrastis, ......................2 fluid ʒ;
Compound tincture of benzoin, ...................2 fluid ʒ;
Carbolized olive (carbolic acid, 5 per cent) or linseed oil, ............ ........................... 1 fluid ℥.

Mix. Sig.: Shake well before using.

The patient is advised, prior to using this injection, that a desire to have the bowels evacuated will occur as a result of its employment, but that if he will remain quiet upon the examining table the sensation will quickly pass away. This uncomfortable feeling is probably due to the alcohol in the fluid extracts as much as to the action of the other ingredients of the formula.

Upon the first visit, if the skin has a very harsh and dry appearance, Adler paints the entire surface around the anus, for several inches outwards, with a strong solution of silver nitrate (a saturated solution). If any break in the continuity of the skin exists as a result of previous scratching, a little of a two-per-cent cocain solution, applied to the abrasion or abrasions, will prevent the suffering incident to the use of the silver salt. In his experience the use of a strong silver solution is not nearly so painful, under the circumstances surrounding its use in the class of cases under consideration, as the weaker solutions.

The application of the silver may have to be repeated two or three times before the desired effect is obtained—not oftener, however, than every third day. By its use Adler desires to have the skin become supple and healthy looking. So soon as the silver has dried, and from the first visit and thereafter, he smears over the anus and the cutaneous surface of the parts, for a distance of about two inches around the orifice, the official citrine ointment or unguentum hydrargyri nitratis. The ointment he uses in its full strength. Over the salve he places a wad of absorbent cotton, the quantity of cotton varying with the patient's wishes and comfort. The dressing is kept in place with a T-bandage. The patient usually comes in the morning for his treatment, and he is advised to wear the dressing all day and over night. If the itching should annoy him during the night, he is directed to bathe the anus with hot water, as hot as can be borne, but under no circumstances is he to rub the parts. He is also told that the application of the hot water will momentarily increase the itching, but that he is not to scratch. After he has used the water he is directed to employ either a solution of blackwash (lotio nigra), or, what is better in some cases, calomel ointment, either of which is to be applied locally to the affected parts. Prior to coming to the office for the next treatment he may wash the parts with Castile soap and hot water, but this is not essential as a routine practice. In bathing the parts no rubbing is to be permitted.

For the first two or three weeks the patient is seen every day; then every other day for a like period or longer, frequently for six weeks, after which time once or twice a week will suffice until he is satisfied that the disease is conquered. Usually this treatment consumes, in its entirety, not over six months. In no case should a definite promise be made to a patient as to the length of time the treatment will consume. Such a course, as a rule, leads to disappointment and dissatisfaction. It is perfectly proper to cite the experiences had with other patients with like affections as examples of what may reasonably be expected by the patient about to undergo treatment.

Adler is also in the habit of warning patients that at any time during the course of treatment the itching may return suddenly and be as severe as at any time prior to coming under observation, but that this must not be deemed a bad omen, as such occurrences were often experienced, but that they had no special significance.

Sometimes, during the use of the nitrate of mercury ointment, the anus and adjacent parts become sore, presumably from the patient scratching, frequently while asleep. Under these circumstances the ointment will have to be discontinued for a few days; during the interim he employs the calomel ointment, in the same manner as directed for the use of the other mercurial salve. The author has never witnessed any bad effects from the use of mercury, such as salivation, etc.

Adler concludes by simply adding that he has treated quite a large number of patients with this disease and by this method, and that he has yet to experience a single failure to effect what thus far has seemed a radical cure. Some of the patients have been treated as long ago as five years, but in no instance, so far as he has had knowledge, has there been any marked return of the trouble. In some few of the cases a patient has returned, a year after discharge, for three or four treatments, owing to some slight sensations experienced about the parts, which they were afraid might portend a return of the old trouble, and wisely deeming that a stitch in time saves nine, sought advice before the much-dreaded affection had an opportunity to obtain another foothold.—*Therapeutic Gazette.*

---

### SYMPTOMATOLOGIC DIAGNOSIS OF VALVULAR OBSTIPATION.*

Obstipation is that form of impaired defecation which is due to the presence in the rectum of an organic obstacle to the descent of the feces through it.

If it be the function of the normal rectal valve to beneficently retard the descent of the feces, it is obviously true that it may be the especial property of the valve in certain other than normal conditions to maliciously obstruct the descent of the feces.†

The patient is the subject of more or less chronic obstipation. He sometimes makes frequent partially successful attempts at defecation daily, but may experience an unrequited desire for food. The patient acquires the reprehensible physic-habit. In time the periods of obstipation are interrupted by short periods of diarrhea. There is an ineffectual straining at stool except for fluid feces. Later the diarrhea occurs with greater frequency, and ultimately long periods of diarrhea may ensue which are interrupted by a transitory constipation and obstipation. All these symptoms may be accompanied by increasing degrees of flatulence and borboryg-

*Read by Thomas Charles Martin, M.D., Cleveland, at a meeting of the Ohio State Medical Society at Columbus, May 10, 1900.

†Obstipation, a Practical Monograph on the Disorders and Diseases of the Rectal Valve. Thomas Charles Martin, Ph.D., M.D., Philadelphia Medical Publishing Company.

mus; and from time to time the patient is subjected to attacks of intestinal autointoxication, and finally he becomes neurasthenic. On account of the especial nonsensitiveness of the rectal valve the patient's sufferings are not uniformly referred to this region by himself, but in many instances, however, the intelligent patient is prepared to present his physician with a ready-made diagnosis of rectal obstruction. Ultimately the symptoms of intestinal obstruction may become pronounced, and if the patient be unrelieved the disease proceeds to a fatal termination. Symptoms of pain, aching in the sacral and iliac regions, hemorrhage, catarrhal or membranous proctocolitis, prolapse, hemorrhoids, fistulas, etc., may be the signs of concomitant sequels of the obstructing valve, and these may embrace the entire proctica.

The *position* of the obstructing valve and the *character* of its disease may be determined from the patient's symptoms and, I may add also, that one skilled in proctoscopy may, on inspection of the rectum, detail to the patient the history of the case without a word having passed on the subject.

1. Valvular obstruction *below* the rectosigmoidal juncture.

2. Valvular obstruction *at* the rectosigmoidal juncture.

3. (a) Anatomic coarctation or congenital juxtaposition of the rectal valves.

(b) Congenital hyperplasia of the rectal valve.

4. Hypertrophy of the rectal valve.

5. Fibrosis of the rectal valve.

1. Valvular obstruction *below* the rectosigmoidal juncture is characterized by straining at stool for the passage of solid feces, and, later on, when the obstruction has developed to a very considerable degree, even fluid feces and enemas are voided with increasing difficulty. After defecation the patient continues to experience a sense of the presence of feces in the lower rectum. The form or mold of the fecal mass is not significant of the presence or absence of a hypertrophied valve for the reason that the mass voided may have resided in a capacious rectal chamber below the obstructing valve and here have been molded to a size many times the diameter of the contracted valve-strait through which it has previously passed. The depth and density of the rectal valve constitute the obstruction rather than does the contraction of the valve-strait—that part of the lumen of the bowel between the free margin of the valve and the opposite rectal wall. However, in cases of annular stricture of the rectum (which are invariably built on the foundations provided by the rectal valve) the contracted valve-strait constitutes an additional but secondary factor in the obstruction.

2. Valvular obstruction *at* the rectosigmoidal juncture is characterized by long intervals between the acts of defecation; by an occasional passage of a considerable quantity of feces without straining, and by tenderness and a sense of fullness in the left iliac fossa and lower abdominal regions; by gaseous distention of the sigmoid and colon and by a sensation of sudden arrest to the passage of gas at a point between the promontory of the sacrum and left iliac spine. There is not experienced that sensation which compels the patient to recognize an impending necessity for defecation, nor that touch-sense in the rectum which prompts the patient to such attempt. An intelligent patient understands the necessity for emptying the lower bowel, but soon appreciates that the mechanism of defecation is sufficiently disordered to make natural attempts practically useless.

3. (a) Anatomic coarctation or congenital juxtaposition of the rectal valves is characterized by difficult defecation of solid or semisolid feces in infancy and childhood. If two rectal valves develop in such a degree of anatomic propinquity that the pressure of the feces on the proximal valve will put the free border of that valve upon and across the next lower valve, there is erected at one point an almost impassable barrier to the descent of the feces for the reason that the interlocked valves unite to form a diaphragm which temporarily walls off the rectal chamber occupied by the feces from the next lower empty chamber. (b) Congenital hyperplasia of the rectal valve which is due to an embryonic overgrowth of the mesoblastic layer of the blastoderm, is characterized by the same symptoms as anatomic coarctation of the valves.

4. Hypertrophy of the rectal valve is characterized by the almost sudden establishment of obstipation. The condition is initiated by a sense of gentle aching and moderate heat in the sacral region, by aching down the thighs, and by discharges of a small amount of viscid mucus. The symptoms are significant of a rectitis which results in infiltration of lymph into the structure of the valve and the rapid organization of the plastic exudate into fibrous tissue. There may be the history of partial improvement in defecation lasting for but a short period, which is followed by the recurrence of the symptoms just described, and exacerbation and amelioration alternate until ultimately chronic obstipation obtains as a product of the recurrent attacks of rectitis.

5. Fibrosis of the rectal valve is characterized by a very gradual development of the obstipation which has its causation in the degeneration of the muscular elements of the valve and the formation of fibrous tissue, which abnormal increase of this element renders the valve rigid to such a degree as to prevent its effacement under the pressure of the forces of defecation.

Valvotomy cures.—*Cleveland Journal of Medicine.*

---

### INDICATIONS FOR OPERATIONS UPON THE LIVER AND BILIARY PASSAGES.

Pantaloni's recently issued work upon this subject (*Chirurgie du foie*, etc., Paris) gives, among much other categorical information, the indications for very many procedures of surgical intervention, which indicate the rapid growth of our knowledge upon these subjects.

Puncture of the liver may be either exploratory or therapeutic, the latter being largely restricted to hydatids. Aseptic exploratory puncture is used as a routine diagnostic procedure, being contraindicated only in suspected malignant diseases, in which exploratory laparotomy is to be preferred.

Intrahepatic injection is indicated in the treatment of hepatic abscess and hydatids.

Laparotomy, classified according to locality into parahepatic, transpleural and lumbar, is done both for exploratory and therapeutic purposes. The former is practiced in trauma of all kinds, whether recent or old. The latter has been employed with success in a few cases of cirrhosis, tuberculosis and syphilis, and malignant disease has even been improved by this intervention. *Advanced* cancer, however, is usually made worse by laparotomy.

Thermocautery of the liver, however performed, has been employed only to check hemorrhage, in connection with other operations.

The parahepatic tamponade is often employed in connection with laparotomy, as a mode of drainage whenever the latter is indicated.

Intrahepatic curettage is indicated in abscess, hydatids and syphiloma, in connection with laparotomy, hepatectomy, etc. Suture of the liver is practiced in trauma, either accidental or operatory, also in hepatopexy, resection, hepatotomy, etc.

Hepatotomy is done sometimes for exploratory purposes alone, with the object of locating deep hydatids, syphilomata, etc. For curative purposes (aside from resection) this operation has been practiced to divide double monstrosities (xipophagi), and in connection with liberating hydatids.

Hepatotomy is an operation practiced in connection with the treatment of abscesses and cysts of the liver, in order to secure drainage and permit of treatment of the interior wall of the cavity; also to anticipate the extension and internal rupture of the accumulations of fluid.

Partial hepatectomy is done in connection with the treatment of wounds, hernia of the liver, numerous infectious granulomata (syphilis, tubercle), tumors of all kinds.

Hepatopexy is practiced for floating liver.

Hepatic phlebotomy has been done in various congestive and inflammatory affections of the liver and biliary passages.

Radiography of the liver is practiced for suspected gall-stones.

Parabiliary laparotomy is indicated as a step in the numerous operations practiced upon the biliary passages and gall-bladder.

Catheterism of the biliary passages is either exploratory or therapeutic in aim, and is of general applicability.

Injection of the biliary canals is done for the purpose of asepsis in connection with other operative procedures. Under this head the insufflation of air may be mentioned.

Drainage of the biliary passages is done in connection with other procedures, and has for its aim the lowering of the tension of the bile, preventiou of infection, and is, in fact, a step of wide applicability.

Cholelithotripsy has been done a few times as a step in the removal of gall-stones.

Simple incision of the gall-bladder, cystic duct and common duct is a step which must be taken in most operations for gall-stones. Lithectomy is a term which denotes the actual ablation of the stone after incision.

Extraperitoneal cholecystostomy is an operative procedure of wide applicability in affections of the biliary passages.

Puncture of the gall-bladder is resorted to only in cases of necessity for exploratory purposes. This maneuver has not much of a field in therapeutics. Cholecystotomy, on the other hand, is an operation which must be performed in every case of biliary infection, in calculus of the gall-bladder or cystic duct, and in drops of the gall-bladder.

Cholecystostomy is one of the best known of all operative procedures about the biliary passages. The leading indication is in biliary infection, with or without the presence of lithiasis, including hypertrophic biliary cirrhosis, angiocholitis and cholecystitis.

Cholecystectomy, or ablation of the gall-bladder, has been done for tumors, including incipient carcinomata, and for persistent biliary fistulæ (in connection with entero-anastomosis), certain traumatisms, inveterate biliary lithiasis, certain advanced forms of cholecystitis, etc.

Cholecystenterostomy (anastomosis of gall-bladder and intestine) is an extensive resource in certain cases of obstruction of the common duct from tumors, calculi and stricture, and in fistula of the gall-badder.

Dilatation of the biliary passages and plastic operations (cholesyringectomy) are employed in connection with the treatment of fistulæ.

Numerous operations are performed upon the cystic duct in connection with the removal of gall-stones (cysticolithotripsy, lithectomy, cysticotomy).

Cysticectomy, or resection of the cystic duct, has been done in connection with an operation for calculus.

Cysticoenterostomy has recently been performed for obstruction of the common duct.

Many operations upon the common duct have been performed, most of them within comparatively recent years. Explorative duodenotomy for examination of the common duct, choledocholithotripsy, choledocholithectomy and choledochotomy have all been employed for calculi within the common duct. Choledochectomy and choledochoenterostomy have also been performed—the first unsuccessfully, while the latter, in which anastomosis buttons are used, is pronounced to be one of the most delicate and brilliant of all intra-abdominal operations.

Operations entirely similar to those performed upon the common and cystic ducts have also been carried out in connection with the hepatic duct (hepaticotomy, hepaticolithectomy, etc.), and upon the dilated biliary radicles (cholangiostomy, etc.); the indications were naturally connected with the presence of calculi, infection, etc.—*Medical Review of Reviews*.

---

### IN LIGHTNING VEIN.

"In lightning vein," we may well include the following statements from a recent optimistic paper in one of our exchanges on "The Therapeutic Value of Static Electricity," read at the West Baden Summer Meeting of the Mitchell District Medical Society:

"You may expect to cure a great majority of your cases of amenorrhea by general electrization and shock. For painful menstruation I have never had such beneficial results as I have had from static electrization. I tell my patient that at the first appearance of pain to come to my place. She does. If she is able I have her sit in a rocking-chair on the positive insulated platform, with negative pole grounded to water and umbrella overhead for one to five minutes. Then I give her local drawing from gas plant in right and left iliac region until relieved, which time is from three to fifteen minutes. The pain has subsided and my patient is to sit around or lie on lounge for the balance of the day or night, as I am prepared to keep them.

"Malaria is successfully cured by electricity with but few treatments, probably on account of the ozone which the system receives. On high mountains we see no malaria, and in swampy flat countries we have plenty, because there is a scarcity of ozone in miasmatic countries.

"We can abort an incipient cold, ease the pain of an abscess, make more rapid the convalescence from fever, brain fag, bronchitis, gastralgia, functional and real disease of the heart, heat prostration, and I might mention at least one hundred other troubles where electricity will do as much to relieve as any other medical treatment. As to how electricity does all these wonderful things is not so plain.

I should not be surprised if in a few years electricity will be given fifty times in a hundred where medicine is needed."

And thus ended the first lesson in the manifold uses of static electricity. We recall equally optimistic statements in regard to the 'possum's tail as a medical panacea. They are quoted from Marcgraves's History of the Spanish-American Colonies of some three centuries ago.

"The tail of this animal (Didelphys Virginiana) is a singular and wonderful remedy against inflammation of the kidneys, and if the tail be chewed and laid on a part in which thorns have been thrust it extracts them, and I believe in all New Spain there is not to be found another remedy as useful in so many cases."

The 'possum's tail, like electricity, is also applicable to genital and maternal disorders: "Excitat veneram, et generat lac; medetur colicis doloribus, prodest pareintibus, et accelerat partum, promovet menses." Of course for these medical applications of the 'possum's tail a rocking-chair or lounge could be provided, and the patient could "sit around" or lie on the lounge for the balance of the day or night, less if need be, just as in the application of static electricity. The absence of hairs on the 'possum's tail does not, as far as we have investigated, detract from its medical virtues; indeed this singular phenomenon may have enhanced its virtues. As is well known, the tail is hairless, for the hairs were use by

Ham on the ark for banjo strings (according to that high evolutionary authority, Irwin Russell, in *Scribner's Monthly*, January, 1878).

Now Ham the only nigger that was runnin' on the packet,
Got lonesome in the barber-shop, and c'u'd'n't stand the racket;
And so for to amuse heself, he steamed some wood and bent it,
And soon he had a banjo made, de fust dat was invented.

He strung her, tuned her, struck a jig, 'twas "Neber min' de wedder,"
She soun' like forty-leven bands a playin' all togedder;
Some went to pattin', some to dancin'; old Noah called de figgers;
An' Ham, he sat and knocked de tune, he happiest of niggers!

Now sence dat time, its mighty strange, deres not de slightest showin'
Ob eny hairs at all upon de 'possum's tail a-growin';
An' curis too, dat nigger's ways, his people nebber los 'em,
For where you finds de nigger dar's de banjo an' de 'possum!"

The above explanation of the absence of hairs from this potent remedy may be of interest to those who wish to apply it intelligently, for as with electricity, "how the 'possum's tail does all these wonderful things is not so plain," and we can not know too much of the action of our remedies.

Static electricity, no doubt, has its uses, and so does the 'possum's tail, as our quotation shows. But we should be conservative in the use of such powerful remedies and not expect too much from them. "Chewed 'possum's tail," is no longer used as an aphrodisiac, a parturient, galactagogue, or even to extract thorns from the foot; for the latter, "chewed tobacco" has been found to be equally "drawin'," by the country people, and it is certainly a better antiseptic. But "the old order changeth," and static electricity is now the prevalent hoodoo among the suggestionists.—*Indiana Medical Journal.*

---

### DANGERS OF VAGINAL IRRIGATION.

Theilhaber (*Münch. med. Wochensch.*, June 12, 1900) recently read a paper with the above title before the Munich Gynecological Society. Most physicians are aware, he says, of the dangers of intrauterine irrigation, but few know that even the vaginal douche may be productive of very unpleasant consequences. He relates a case in point which occurred in his practice as far back as 1886.

The patient had an erosion of the vaginal portion for which she had been using a douche. While irrigating her vagina she was seized—in the midst of apparently perfect general health—with severe pains, malaise, vomiting, etc. She discontinued the douche and sent for the author, who found a small, frequent pulse, tympanitic and sensitive abdomen, pallor, etc. The syndrome was that of acute pelvic peritonitis which yields to the steady employment of an ice-bag with bed-rest for a number of days.

Since 1886 the author has had four other similar cases in his practice—somewhat less severe in type than the first, but well-defined. He admits that this accident is infrequent in occurrence and implies that some authorities might dispute the existence of a casual connection between the vaginal douche and the symptoms which followed thereupon. Nevertheless both physician and patient occasionally note the sequence of douche and pain. Certain authors (Hegar-Kaltenbach) allude to the possibility of the douche causing chills, swooning, nausea, etc., under certain conditions. The literature of the subject shows that cases of this sort have been reported by E. Martin, Ebell, Kock, Madden, Engelmann, Spaeth, Thomas (U. S. 1877), and Kisch.

A careful analysis of the subject shows that accidents following vaginal douching fall under several categories, as follows:

1. In sensitive women, too hot or too cold water may lead to shock, chill, nausea, etc.

2. If peritonitis is already present, the douche may cause an aggravation, especially if the intravaginal pressure is raised (which occurs if the ostium is unduly narrow, or if the fluid is injected under too much headway).

3 Very rarely the douching fluid has entered a vein in certain cases of cancer.

4. The majority of accidents, however, have been due to the entrance of the irrigating fluid into the patent os by reason of the engagement of the nozzle of the syringe, etc., in the latter aperture. The olive-shaped end of the canula prevents the return flow of fluid, the uterus is dilated—as was demonstrated by Beuttner in 1897—and pain, with shock, emesis, etc., are produced.

5. There can hardly be a doubt that the irrigation fluid may at times penetrate the tubes and enter the peritoneal cavity. The state of affairs then becomes the same as that which follows the same accident in connection with intrauterine douching. It can readily happen that infectious particles are carried up from the vagina or uterus into the peritoneal cavity—especially is this true of gonorrhea.

How may we so conduct vaginal douching as to prevent the possibility of these accidents? In the first place, certain apparatus which carries with it danger of raising the intravaginal pressure or of chance lodgment of the canula into the os must be done away with. The author recommends a vaginal nozzle in which the opening is not situated at the extreme end, but behind the olive-shaped expansion. Rock, Lehmann and Ahlfeld have recommended similar modifications, all of which tend to prevent the chance introduction of the douche-water into the os uteri, and also to favor the prompt return of the fluid and thereby favor the prevention of over-distention of the vagina—*Medical Review of Reviews.*

---

### THE ABOLITION OF THE ARMY CANTEEN.

One of the most misguided bills which has been introduced into Congress this session is that abolishing the army canteen, and it is pretty generally condemned by army officers and surgeons. The idea of abolishing the army canteen as an aid to compulsory teetotalism is a mistaken one. The canteen is in reality the enlisted man's club. Here he may buy beer and liquors, lunch, cigars and smoking tobacco, and numerous other things necessary to a soldier's life at an army post. Everything sold at the canteen is purchased under the supervision of an officer, and so it is likewise sold. Purchases are made by check, and thus an accurate account is kept of what each man gets. Thus, the quantity is known. As to quality, that is known, too, as the best. From a purely temperance view-point the canteen is a safeguard. If it is abolished the men will get liquor outside their posts, and get any quantity of a very uncertain quality too, and in some one of the many saloons which spring up in the immediate vicinity of a post.

Naturally, there is considerable profit from the conduct of a canteen at a fairly well-conducted post, and this is equally divided between the companies and the hospital corps.

Army surgeons of wide experience, in writing on this subject, are almost unanimous in saying that there is less sickness at a post where a canteen is operated than where there is none, and that drunkenness is much more common among the men in the latter case.

In this connection, a letter written to the New York Sun by an army officer will be interesting. In part he says:

The advocates of this measure seem to assume that their ideal fits an actual state of affairs that does not exist. The fact that nine men out of every ten drink lager beer and will have it, or some substitute in lieu of it, is the controlling factor in the question, and it is impossible to legislate this desire from their physical make-up.

The question then comes down to a choice as to whether it is better to allow the soldier a limited quantity of good lager, dispensed and consumed under military restrictions, or to drive him to the dives which surround the approaches to every military post, there to get vile liquor, and to be exposed to the many evil influences that thrive in such places.

The writer lived the first eighteen years of his life in "prohibition" Portland, Me.; for the past ten years he has served at various posts from New York to San Francisco, and from the Columbia River to the Gulf of Mexico, and can say from that experience that he has never seen a more drunken city in the United States than "prohibition" Portland." The chief result of prohibition is to drive beer out and the vilest of vile whisky in. This will be the result to the army if this pernicious bill becomes law.

After the Spanish war the writer was stationed at Washington Barracks, D. C., where temporarily there was no canteen. During that winter the guardhouse was full to overflowing as the result of indulgence in 4½ Street whisky.

The decrease of drunkenness in the army, and the improvement in discipline, temperance and decency during the past ten years has been very marked, and is due entirely to the good influence of the Post Exchange.

The Post Exchange is an enlisted man's club, and comprises, besides the bar, a lunch room, billiard room, reading-room and store. The patronage of these, at this post, is about three-fifths of the bar, and the soldier receives the benefit of low prices impossible except for the sale of beer.

Furthermore, the profits of the Exchange are divided monthly among the messes of the men, and greatly improve the fare of the soldier. Withdraw this help and put the soldier on the ration, *i. e.*, "government straight," and disaffection, desertion and drunkenness will result.—*The American Medical Quarterly.*

---

### LAPAROTOMY FOR ABDOMINAL WOUNDS IN WAR.

Mr. Frederick Treves (*British Medical Journal, May 12th*), in a paper on The Wounded in the Present War, recently read before the Royal Medical and Chirurgical Society, enumerates the circumstances in favor of operations as follows: 1. If the patient is seen before seven hours, which is not an unreasonable limit. 2. If the patient has had a short and easy transport. They had many of those. 3. An empty stomach. He would say that in some few antero-posterior wounds of the abdomen above the umbilicus it is a *sine qua non* that it should be a case with an escaped bullet, especially when associated with extensive hemorrhage. Then he deliberately believes that a patient ought to be operated upon. It might be thought that such cases would be very common; but, in fact, they are very uncommon. The circumstances against operation are: 1. If the patient is not seen till more than seven hours after the wound. He does not say that this is an absolute bar, but it is one argument against operation. 2. If there has been a long and arduous transport. 3. If he has been wounded soon after a meal. That is very improbable. 4. He would exclude all cases of transverse or oblique wounds above the umbilicus, because it is practically impossible to do all that is required. He did some operations in such cases, and found six, eight, ten, twelve openings in the bowel, and when he had seen to these he had six holes in the mesentery, and very likely wounds in the liver. He referred to transverse or oblique wounds. 5. He excluded all cases of retained bullet. We can see the point of entrance, but where is the bullet? It may have gone this way or it may have gone that way; we do not know anything about it. We can not in the field embark on any of those operations where we can bring out the viscera in the same way as we might post-mortem. It may be very well in a hospital, but it cannot be done in the field. He would not touch such a case. 6. He would exclude all cases of wound of the liver, of the spleen and of the kidney. There were such cases in large numbers. A certain famous officer had a shrapnel bullet go through the liver and through the right kidney. He had come home here and was now quite well. 7. Most wounds below the umbilicus did all right if they were left alone. 8. Lastly, he would not operate upon a case in which he thought the colon was implicated alone (he might except the transverse colon), because the cases did very well if they were left alone. But with an antero-posterior wound with an escaped bullet, which was a *sine qua non*—that was to say, if where it went in and where it came out could be seen—he believed he had learned that the bowels did not move after they had been struck.—*New York Medical Journal.*

---

### CATARRHAL DEAFNESS.

Dr. Dunbar Roy, of Atlanta (*The Medical Times* for June), makes a plea for more minute study of every case of catarrhal deafness, and deprecates the habit of routine treatment. He thinks that stenosis of the Eustachian tube causes many, but not all, cases of catarrhal deafness. Undue patency may exisit as an etiological factor in some cases. Where stenosis does exist, he advocates medications applied direct to the membrane of the tube and internal ear through the catheter, and in cases of distinct stricture he used whalebone bougies to overcome fibrous contraction. He has not had as good results from rapid dilation by means of electrolysis as have been reported by A. B. Duel, of New York (*Medical Review of Reviews* for May, page 334).

As to prognosis, he considers it to depend upon:

1. Age of the patient.
2. The pathologic condition of the nose and nasopharynx.
3. Duration of the deafness.
4. Condition of the Eustachian tube.
5. Mobility of the drum and ossicles.
6. General health of the patient.

As to treatment locally, he much prefers liquids, such as solutions of iodin and menthol in albolene, to the injection of vapors. Pneumatic massage he has found useful where the drum is retracted, if the accompanying ankylosis of the ossicles is not too firm to permit of movement. The simple method of suction through Siegel's speculum he considers has given him as good results as more complicated and expensive apparatus.

His summary of treatment is as follows:

1. See that the nasal cavities and nasopharynx are placed in as healthy condition as possible by the treatment of all catarrhal states and the removal of all obstructions to free respiration.

2. See that the Eustachian tube and the middle ear are medicated at proper intervals in addition to the inflations.

3. Render the drum and ossicles as pliable as possible by some system of massage.

4. Don't forget the general health of the patient.—*Review of Reviews.*

---

### SURGICAL FANATICISM.

Such is the title of an article by Dr. Ap. Morgan Vance, of Louisville, read before the Kentucky State Medical Society at Georgetown, May 9-11, and published in the *Louisville Monthly Journal* for August. Illustrative operations, which Dr. Vance vigorously satirizes and condemns, are Kraske's resection of the sacrum to make growths of the rectum or uterus accessible, advocated by Dr. Montgomery, of Philadelphia, and carried out by an enthusiastic post-graduate who desired to shine as a great surgeon, with the result of three cases and three deaths. The rule of the greatest good to the greatest number does not justify this operation.

Second, the author castigates the fanatics who operate in forlorn cases of breast cancer, and particularly recurrent ones. His case in point was dissected twice, against the advice of Dr. Vance, in an Eastern institution where radical breast work is done, dying shortly after the second operation. These are cases of "surgical fanaticism."

Dr. Vance's third attack is on the advocates of supra-vaginal hysterectomy for cancer, with removal of the adjacent lymphatics, a method of work "so void of common sense" that "it is too absurd to mention." "This sort of thing is sheer nonsense, and should be frowned down by all more conscientious and less enthusiastic surgeons."

Edebohl's operation for examination of one or both kidneys through a lumbar incision is quoted at length from his paper before the New York State Medical Society, January 28, '98, and is regarded by Dr. Vance—himself a kidney surgeon—as a grave operation and practically impossible of good results.

The circumcising fanatics receive due attention. The elittoris and foreskin were made for a purpose and not to be mutilated or removed. Early reflexion in childhood or infancy is all that is required; then the glands will take care of themselves. "These faddist should be more conservative.

"Twelve operations and twelve funerals" is the record for ligation of the abdominal aorta. The case of Wilson & Keen is cited.

Thus endeth the story of Ap. Morgan Vance. There will no doubt be plenty to answer him. But the outburst against extreme surgical procedures in cancer led by McMurtry, of Louisville, as against the teaching of the Eastern medical centers, has incited many papers. Indeed, there is a plethora of papers on "Conservative Surgery" in present periodical literature.—*Indiana Medical Journal.*

---

### POINTS IN SURGERY.

The following "catchy phrases" are from the pen of Dr. William V. Morgan, of Indianapolis:

1. A soft chancre burneth away fast.
2. An uncut felon should be considered a felony.

3. For lacerated perineum septic repair is worse than neglect.

4. "Milk fever" belongs to the suckling stage of obstetric practice.

5. "Delayed shock" means either hemorrhage or sepsis; decide quickly and act boldly.

6. The golden rule for the passage of urethral sounds or catheters is, "Begin with the larger size."

7. Prolapsed funis calls for podalic version. Funis repositors should be left in repose. Delay means death.

8. Chloroform given near an open flame is likely to be decomposed into irritating and poisonous vapors.

9. In cases of obstruction of the bowel, with stercoraceous vomiting, lavage of the stomach, both before and after surgical interference, will greatly enhance the patient's chances of recovery.

10. One thing about which it is good to be "cranky" is adherent prepuce. Let no such pathologic condition escape you unrelieved. In convulsions of children, male or female, the prepuce should always be examined. Ten good digits in two good minutes suffice to overcome the resistance of the most adherent prepuce that was ever hung to a boy.

11. A 10 per cent. solution of antipyrine is a sovereign remedy for vesical hemorrhage. From four to eight ounces may be allowed to remain in the bladder for thirty minutes.

12. Don't be too quick to promise a perfect result after dislocation at the shoulder. The circumflex nerve passes closely around the surgical neck of the humerus, and often takes serious and lasting offense at the traumatism. Paralysis of the deltoid prevents abduction of the arm, permits gradual elongation of the capsular ligament, and recovery from it is usually slow and incomplete; hence the wisdom of a lagging prognosis.

---

### NEWS ITEMS.

From the newsy *Medical Age* we learn that Louisiana has the only home for lepers in the United States; that Harvard Medical School graduated an even 100 students; that there are 8,000 medical students in the German universities; that Dr. W. W. Keen has raised $50,000 for the library of the College of Physicians in Philadelphia; that the medical schools of the Northwestern University of Chicago for men and women will be continued as separate institutions; that Lord Lister, the father of antiseptics, will give the third biennial Huxley lecture on "Recent Advances in Science and their Bearing on Medicine and Surgery," at Charing Cross Hospital, London, October 2d (the first lecture was by the great physiologist, Michael Foster, and the second by the eminent pathologist, Professor Virchow); that a male child three months old was shown at the Johns Hopkins Hospital Medical Society with a boneless tail two and one-half inches long, which retracts at the tip when the child cries; that the xiphopagous twins, 8 years old, Rosalina and Maria, were successfully separated recently by Dr. Alvara Ramos, of Rio Janeiro, and that one died a few days thereafter; that in Bombay Presidency 20,000 cases of cholera occurred in June, with 12,000 deaths. The following story of Sir William MacCormac, the president of the Royal College of Surgeons, is reliable: "Often to save time when studying in his laboratory, he used to have a light lunch served there. Once his assistant heard him sigh heavily, and, looking up, saw the doctor glaring at two glass receptacles on his table. 'What is the matter, doctor?' he was asked. 'Nothing in particular,' was the reply; 'only I am uncertain whether I drank the beef tea or that compound I have been working on.'"—*Indiana Medical Journal.*

---

### DETERMINATION OF SEX.

The conclusions of the latest German writers are that the sex is already decided in the ovary, as has been conclusively shown for bees and certain lower forms of life. The only means by which the determination of sex can be influenced is by the nutritional processes in the ovary. Disturbances in the ovary in this line, dating possibly from fetal or infant life, seem to determine a preponderance of male ova, while abundant, normal nutritional processes favor the production of females. Ploss has noted a coincidence between the higher prices of provisions and the larger number of boys born. Schenk, on the other hand, considers the female offspring the evidence of nutritional disturbances, as several mothers of boys gave birth to girls after symptoms of diabetes were first observed, which was the origin of his famous method of sex determination.—*Journal of the American Medical Association.*

---

### CHICAGO DRAINAGE CANAL DIMINISHES TYPHOID FEVER.

The Health Department Bulletin of Chicago records a great reduction of typhoid since the canal was opened into the Illinois river, thus taking away the sewage from the Chicago supply of Lake Michigan water. In five months the reduction amounts to 330 fewer deaths than the average for the preceding ten years. At $5,000, the legislative value of life, the saving is $1,100,000. Chicago is phenomenally free of typhoid. The April Bulletin shows twenty-three deaths out of a population of 1,750,000. In May there were but nineteen deaths. Indianapolis, with a population of 150,000, lost three cases of typhoid in April and four in May, very nearly the same proportion as in Chicago. The deaths from cancer in both cities are about four times as many as from typhoid.—*Indiana Medical Journal.*

---

### IMPERIALISM AND PORTO RICO SMALLPOX.

One of the most noticeable of the acts of oppression for which the imperialistic government is responsible is the refusal to let the people die of smallpox. The year before the Americans came smallpox carried off 522 natives, and eighteen months ago 3,000 cases were known to exist. This afforded a pretext for the imperialists to manifest their power by ordering the vaccination of the entire population, and 790,000 of 900,000 were successfully vaccinated. No death from smallpox has occurred in the last eight months. The imperialists have taken away from the natives the privilege of having smallpox "without the consent of the governed."—*Indianapolis Daily Journal.*

